# Digital Health Literacy Among Immigrants in Spain: Implications for Health Equity and Policies

**DOI:** 10.3389/ijph.2025.1608436

**Published:** 2025-10-24

**Authors:** Inés Rey Hidalgo, Ana Fernández-Feito, Sarah Wamala Andersson, Cristina Fernández García, Beatrice Avagnina, Marta María Pisano-Gónzalez

**Affiliations:** ^1^ Departamento de Proyectos Europeos, Fundación Para el Fomento en Asturias de la Investigación Científica Aplicada y la Tecnología (FICYT), Oviedo, Spain; ^2^ Instituto de Investigación Sanitaria del Principado Asturias, Oviedo, Spain; ^3^ Departamento de Medicina, Universidad de Oviedo, Oviedo, Spain; ^4^ Division of Public Health Sciences, School of Health, Care and Social Welfare, Mälardalen University College, Västerås, Västmanland, Sweden; ^5^ Servicio de Salud del Principado de Asturias (SESPA), Oviedo, Asturias, Spain; ^6^ Consulta Europa Projects and Innovation SL, Las Palmas de Gran Canaria, Spain; ^7^ Consejería de Salud del Principado de Asturias, Oviedo, Spain

**Keywords:** digital health literacy, immigrants, qualitative research, Spain, cultural competence

## Abstract

**Objectives:**

The objective of this study was to qualitatively explore the experiences of immigrants in Spain using the internet for health-related purposes, while identifying the barriers, needs, and opportunities within the context of digital health literacy.

**Methods:**

24 individuals with immigrant backgrounds in Spain participated in semi-structured interviews guided by a participatory framework. Data were analysed using qualitative content analysis.

**Results:**

Immigrants perceive digital health literacy as a valuable tool for empowering them to take a more active role in managing their health. However, socio-economic and cultural barriers such as language limitations and low levels of education were identified. Key needs included improvements in health platforms, particularly regarding access, content and security. Participants advocate for greater involvement from healthcare providers and strategic stakeholders to better adapt services to immigrant communities.

**Conclusion:**

This study provides valuable insights for policymakers, offering evidence-based approaches for inclusive strategies to enhance digital health literacy. It also emphasizes the necessity of policies tailored to the specific health needs of immigrant populations, aiming to reduce health inequalities.

## Introduction

Digital technologies are transforming healthcare and public health systems globally. They hold significant potential to enhance both population and individual health and wellbeing [1]. In the rapidly evolving landscape of healthcare, digital health literacy (DHL) has emerged as a critical skill. The European Region Plan 2023–2030 [2] of the World Health Organisation (WHO) includes the advancement on DHL among its main strategic priorities.

DHL is described by the WHO Action Network M-POHL [3] as “the ability to search for, access, understand, appraise, validate, and apply online health information, and to formulate and express questions, opinion, thoughts, or feelings when using digital devices.” Individuals with higher DHL scores demonstrate better self-management, greater involvement in their healthcare decisions, improved mental and psychological wellbeing, and an enhanced quality of life [4]. Low DHL levels can have significant negative effects on health individuals and increased health disparities [5], limiting their access to healthcare services and information [6]. There is also evidence that the improvement of digital skills among vulnerable populations has a positive impact on their access to healthcare [7].

Just over half of the European countries have developed policies for DHL and implemented a digital inclusion plan in the health sector so new strategies are needed to analyse DHL in the population [8]. Participatory and user-centred design of digital health interventions is crucial to get insights on barriers and needs of the population, mainly vulnerable groups who face specific difficulties to healthcare, such as immigrant populations [9]. Progress on the above-mentioned areas would enable policymakers and healthcare managers to develop more effective policies and interventions, adapting them to meet specific linguistic or cultural needs and enhancing participation [10, 11].

Little literature has been found on the analysis of DHL in Spain and none referred to immigrant populations. Just an eHealth literacy questionnaire has recently been validated in Spain to measure DHL level, but for general population [12]. Further research indicates for adults a need for greater development of digital services tailored to individual needs [13].

All above considered, this study was conducted in the framework of the European project, *Improving Digital Empowerment for Active Healthy Living* (IDEAHL) [14], which was funded by the European Commission (101057477). IDEAHL was aimed to develop new approaches to enhance digital health literacy through the co-creation of a comprehensive and inclusive European DHL strategy. For the generation of such strategy, it was essential to collect information on the barriers and needs of the most representative sectors of citizens, including diverse socioeconomic and cultural backgrounds. Special attention was paid to vulnerable groups, such as immigrant populations. The information gathered during the co-creation sessions informed the European DHL strategy [15] developed in the IDEAHL project.

The objective and main novelty of this study was to qualitatively explore the experiences of immigrants in Spain using the internet for health-related purposes, while identifying the barriers, needs, and opportunities within the context of DHL.

## Methods

The co-creation methodology used in this study was designed in the framework of the IDEAHL project [14]. 14 partners from 10 European countries participated in the design and organization of 140 co-creation sessions involving 19 population groups, such as policymakers, healthcare professionals, and immigrant population, among others. The co-creation methodology emphasizes the importance of involving stakeholders at every phase of the project and is rooted in the principles of participatory design and action research. This methodology is proven to be an essential strategy in addressing the unique needs of vulnerable populations [16, 17].

### Participants

Individuals with immigrant backgrounds residing in the Asturias region (Northern Spain) were invited to participate in the study. The inclusion criteria were: being 18 years or older, possessing basic Spanish language skills, and having lived in Spain for a minimum of 3 months prior to the study.

### Procedure

The research team was supported by two non-governmental organisations (NGOs) in the region, Caritas and the Red Cross, for the recruitment of participants and the organisation of the sessions. These entities are strategic stakeholders within the region, providing a range of services that are designed to support the integration of the local immigrant population into society and participate fully in all aspects of life within the community. These services include, but are not limited to, Spanish language and culture courses, assistance with job searches, and guidance on administrative requirements. The sessions were organized in the facilities of strategic stakeholders in the community, which helped create a sense of familiarity with the participants.

Co-creation sessions: the methodology for the sessions is outlined in Table 1. Upon arrival, all participants were welcomed to the session facilities and reminded that their participation was voluntary. Each participant received a leaflet about the IDEAHL project and was advised to avoid using names during discussions to maintain anonymity. Two distinct participatory techniques were employed in each session to engage participants through diverse methods, enhancing the quality of feedback. This approach followed the IDEAHL methodology.

**TABLE 1:** Methodology of the sessions. Interview guide (Digital health literacy among immigrants, Spain. 2023-2024).

Introduction (25 min) Introduction of the research team and participants (5 min) Description of the purpose of IDEAHL and the concept of DHL (10 min) Description of the session and reminder of anonymity (5 min) Questions and answers (5 min)
Questions (35 min) • What resources are available to you to improve DHL? • What advantages do you find in Asturias/Spain compared to your home country that will help you to improve your health literacy? • What would you highlight about these resources?
Break (15 min)
Gathering (15 min) • Reflection on the ideas that emerged in the first part of the session
Questions (35 min) • What barriers or difficulties do you encounter when searching for, selecting and using health information employing digital resources? • Describe your wish list for improving your health with the use of digital resources?
Summary of the discussion (10 min) • Wrap-up of the main messages discussed (10 min) • Closing, thanks to the participants and hand over of a pen as a gift

Each session was divided into two parts, allowing participants time to reflect on the ideas presented in the first part, enabling them to consolidate their responses in the second part.

The sessions were led by one coordinator and two facilitators. Two strategic stakeholder representatives, serving as co-facilitators, took notes to ensure cultural and target-group sensitivity in the data collection process. A participatory approach was followed. The choice of participatory techniques was made by the facilitators, in consultation with the strategic stakeholders, who offered recommendations based on their prior experience with the participants. In addition, the strategic stakeholders who understand the nuances of participants’ backgrounds, acted as cultural mediators and facilitated culturally sensitive communication, that is, they acted by clarifying expressions that could be confusing. The techniques employed included photovoice [18] and group interviews. A semi-structured interview guide developed in the IDEAHL project was used (Table 1).

Analysis of the information: field notes were collected using a standardized template developed in alignment with the IDEAHL methodology to ensure the quality of the data collected. Immediately following each session, facilitators and co-facilitators engaged in structured debriefings to reflect on the session dynamics, clarify observations, and validate interpretations. These discussions were incorporated into the analysis and contribute to ensuring data quality. Just after the sessions, the strategic stakeholders acting as co-facilitators, and the facilitators met together to revise and validate the information gathered. Three authors with experience in qualitative analysis participated in the analysis. The first author (IRH) analyzed the data in detail to discover saturation and recurrent patterns. This analysis involved rereading the notes several times to become familiar with the content. Themes and sub-themes were generated and indexed along with the written notes of all researchers. A detailed codebook was developed collaboratively prior to coding, outlining definitions and examples for each theme and sub-theme. This served as a reference to ensure consistency and reduce ambiguity during the coding process. To enhance inter-coder reliability, a subset of the data was independently coded by two researchers. Coding discrepancies were discussed and resolved through consensus, leading to refinement of the codebook. Two other authors (MPG and AFF) consolidated 100% of the total coding material to guarantee data quality, consistency, validity, and reliability of results. The reports were analysed using qualitative content analysis [19]. The subtlety of this analytical method helped identify patterns and key perspectives, providing a rich understanding of the barriers and needs related to DHL among immigrant populations. A qualitative data analysis software was used, - MAXQDA^®^ [20].

### Ethical Considerations

The study was approved by the Ethics Committee of Asturias (ref CEImPA 2023.004). All participants received an information sheet and signed an informed consent prior to the session. Anonymity was ensured throughout both the sessions and the data analysis process.

## Results

### Participants’ Characteristics

The characteristics of the participants are described in Table 2. 24 individuals from 12 countries participated in the study. The majority of participants were Spanish-speaking from the Americas (62.5%), while Africa and Europe accounted for 16.7% each and 4.2% from Asia. There were more female participants (75%). Additionally, 58.3% have been in Spain for less than or equal to 5 years.

**TABLE 2:** Characteristics of participants (Digital health literacy among immigrants. Spain. 2023–2024).

Code	Sex	Country of origin	Time in Spain
P1	woman	Venezuela	1–5 years
P2	woman	Venezuela	<1 year
P3	woman	Venezuela	<1 year
P4	man	Venezuela	<1 year
P5	man	Cuba	<1 year
P6	woman	Cuba	<1 year
P7	woman	Cuba	<1 year
P8	woman	Dominican Republic	>10 years
P9	man	Dominican Republic	6–10 years
P10	woman	Dominican Republic	>10 years
P11	woman	Colombia	<1 year
P12 P13	woman woman	Colombia Brazil	<1 year
			6–10 years
P14	woman	Brazil	6–10 years
P15	man	Morocco	6–10 years
P16	woman	Morocco	6–10 years
P17 P18 P19	woman woman woman	Russia Russia Ukraine	1–5 years
			6–10 years
			1–5 years
P20 P21 P22	woman woman man	Ukraine Santo Domingo Cameroon	1–5 years
			>10 years
			<1 year
P23	woman	Bangladesh	1–5 years
P24	man	Guinea	6–10 years

### Qualitative Findings

Overall, all participants use their mobile devices to search for health information online. While they find this information helpful, they all consider advice from their doctor to be more reliable and essential. Many participants value the ability to seek health information online, particularly for disease prevention, as it allows them to access resources on healthy lifestyles. Several mentioned relying on more knowledgeable family members to assist in finding health information online. Participants also appreciate digital media for staying connected with loved ones, which positively impacts their mental health. They express deep gratitude for the support they receive from their community, recognizing that migration presents significant challenges. Overall, they reported having better access to digital resources and healthier habits in Spain compared to their home countries, and they view the Spanish healthcare system as high-quality, generally accessible, and free of charge.

A comparative analysis by sex reveals that women tend to use digital resources more frequently, particularly for topics such as nutrition, healthy lifestyles, emotional wellbeing (e.g., anxiety, stress), specific health conditions (e.g., cancer, migraines), and caregiving. In contrast, men focus more on targeted health concerns like smoking cessation and obesity, as well as practical uses such as fitness routines. Another key difference lies in how information is validated: women often cross-check sources or consult health professionals and family members with medical knowledge, while men are more likely to rely on personal judgment or general trust in the information they find online.

Three main themes, categories and codes were identified (Table 3): significance of DHL, socioeconomic and cultural factors hindering DHL and messages for new health policies.

**TABLE 3:** Themes, categories and codes identified in the study (Digital health literacy among immigrants. Spain. 2023-2024).

Themes	Categories	Codes
Significance of DHL	Perceived usefulness	Help with selfcare and of others Preference for healthcare professionals Understand medical information Contrast of health information Healthier lifestyles Better communicatoin with healhcare professionals
	Type of information	Dietary information Illnesses not diagnosed, emotional and physicial wellbeing Medicines and illnesses diagnosed Home remedies
	Valuable resources	Google Youtube
Socio-economic and cultural factors hindering DHL	Knowledge limitations	Spanish language Low health literacy/education
	Financial constraints	Access cost
	Lack of confidence	Not reliable information
	Immigrants-specific complexity	Not official residence Political conflicts
Messages for new health policies	Digital platforms requirements	Avoid advertisements of private companies Easy-to-use platforms Identification of scientific backing Use images with descriptions Free access to information
	Information requirements	Practical and useful information Easy-to-understand information Trustworthy information Dynamic and entertaining information Adapted to differente population
	Healthcare professional engagement	More involvement in information published on the internet Online consultations Recommendations of reliable sites
	Educational needs	Spanish language Digital skills Information search

### Theme I: Significance of DHL

This theme describes how useful it is for them to be able to search for health information on the internet, for what purpose they search for health information, what type of information they look for and which platforms they use most.

#### Perceived Usefulness

In general, participants said that they prefer to go to health professionals so that the information is always appropriate and reliable,

I find the most appropriate information through the healthcare centre or hospital (man, Dominican Republic, 6 years in Spain).

Regarding the use of digital resources and the increase in health knowledge, most of the participants declared that they find it very useful to learn how to selfcare and how to care for others, family members or friends. One participant mentioned,

I learned to use digital media when I was already here and I communicate with my family through video calls, which I use to pass on everything I have learned about health (woman, Brazil, 6 years in Spain).

In addition, they also find it very useful to understand better the medical information, and also to be able to communicate better with the healthcare professionals, declaring,

I am often forced to inform myself due to a lack of understanding of very technical medical terms, which I do not fully understand, and I need support to understand. By searching the Internet and reading symptoms and diagnoses, I was able to explain to my doctor how I was feeling and was diagnosed and treated for sleep apnea, which I believe may have saved my life (woman, Brazil, 6 years in Spain).

Some participants also explained that they use the internet to contrast the health information they received from family or friends, saying,

I find the internet search very useful. Before I couldn't check the information I was given and now I can look for other options and opinions (woman, Brazil, 6 years in Spain).

Finally, many said that they use it to learn how to have a healthier lifestyle,

The information I obtain helps me to improve my lifestyle, to find ways to feel less tired and more agile. I don’t only look for information when it has to do with illness (woman, Dominican Republic, 20 years in Spain).

#### Type of Information

In terms of the type of information sought by the immigrant population, the majority seek information related to an illness diagnosed by their doctor and prescribed medication. One participant noted,

When I had breast cancer, I found it very helpful to Google search for symptoms, testimonials from others, information on medications and side effects (woman, Brazil, 6 years in Spain).

Another participant, indicated that she looks for information related to emotional and physical wellbeing to help the woman she cares of, saying,

I work as a caregiver for an 80-year-old woman, and I decided to help her with an exercise plan to strengthen her legs. I have been looking for information to improve the mental and physical part. I usually search on Google. Before I started, I met with the children to discuss what I was going to do. I have managed to improve the patient's mental and physical condition (woman, Venezuela, 37 months in Spain).

Many participants stated that they like searching for dietary information and for home remedies, saying,

I find the internet search very useful. I am interested in nutrition, proper nutrition, I can look for other options and opinions (woman, Brazil, 6 years in Spain).

#### Valuable Resources

As for the digital resources they use most, Youtube and Google are the most used, declaring,

I suffer from migraine and I have searched for relaxation methods, home remedies on Google or Youtube and I have looked for information about the medicines I have been prescribed. I found it useful to put it into practice and I also used the internet to check the advice given to me by other people. I regularly look for recipes (woman, Cuba, 3–7 months in Spain).

### Theme II: Socio-Economic and Cultural Factors Hindering DHL

This theme describes the limitations the participants have found to search for health information using digital resources.

#### Knowledge Limitations

Many participants noted that knowledge limitations in terms of Spanish language knowledge and low health literacy or low education, mean an important barrier for them to improve DHL, saying,

Language can be a barrier to digital literacy; speaking different languages is a difficulty. I always bring a translator to medical appointments (woman, Russia, 3 years in Spain).

And,

I don't always find what I’m looking for on the Internet, but I understand that this is due to my low level of education, due to the situation in my country and the lack of opportunities (man, Dominican Republic, 10 years in Spain).

#### Financial Constraints

Financial challenges are pertinent for immigrant population. Some participants expressed concern about the cost of accessing certain information, noting,

I have several applications, but many of them have a cost and that slows me down (woman, Colombia, 3–7 months in Spain).

#### Lack of Confidence

Most participants declared that they do not fully trust the health information they find on the internet meaning that this may be an important barrier for them to increase DHL, saying,

My midwife has also served as a reliable source of information, recommending appropriate websites and social networks where I can ask my questions. My problem, in conclusion, is the reliability of the sources. We would need a health professional to always recommend reliable websites to us (woman, Dominican Republic, 20 years in Spain).

#### Immigrants-Specific Complexity

Participants also referred to an important challenge they face due to their condition of immigrants, which may make them feel anxiety and stress, saying,

I didn't want to leave Russia, we had to leave because of political problems, my husband was in prison. I have a son in Russia, I am afraid for my husband, I live in permanent fear and worry. I watch online relaxation exercises that help me, but I am still scared and quite isolated (woman, Russia, 3 years in Spain).

### Theme III: Messages for New Health Policies

Although some barriers and challenges in using digital resources to look for health information were described earlier, some recurrent needs were identified to improve DHL among immigrant population.

#### Digital Platforms Requirements

Some participants commented on the importance of avoiding advertisements from private companies when using a digital platform and of having free and easy-to-use digital platforms available. One participant indicated,

I would like the information to be truthful and not always try to sell products of certain companies. I want apps with real, practical information, without deception. I want all the information, not to be redirected to other sites or to have to check the information elsewhere. I don't want to be made dizzy (woman, Venezuela, 3–7 months in Spain).

And,

I would like to be able to access the internet and recommended apps for free (woman, Colombia, 3–7 months in Spain).

Additionally, they highlighted the importance of being able to identify scientific origin of the information and the use of images with descriptions, declaring,

I would like to see scientific backing referenced to increase safety (woman, Venezuela, 3–7 months in Spain).

And,

Being able to upload a photo in real time and get an answer (example: dermatitis, upload a photo of the skin and get an answer) (woman, Venezuela, 3–7 months).

#### Information Requirements

As for their needs in terms of the information they are interested in, some participants indicated the importance of including trustworthy, easy-to-understand, practical and useful information, saying,

I would like the information to be easy to understand, without being too medical in language (woman, Cuba, 3–7 months in Spain).

And,

I would like the information to be truthful and not always try to sell products of certain companies (woman, Venezuela, 3–7 months in Spain).

Additionally, the need for the adaptation of health-related information to different types of population was stated as an important element to increase DHL, saying,

Health videos should be adapted to children's ages (woman, Morocco, 10 years in Spain).

#### Healthcare Professional Engagement

Most of the participants expressed their interest in the involvement of the healthcare professionals in the creation of the health-related content available on the internet, which would help increase the confidence on the information available on the internet. One participant noted,

I find the information very useful and the possibility to search on the internet very useful. If the websites were always verified and managed by health professionals, I would use them more (woman, Brazil, 6 years).

The option of online consultations with the healthcare professionals was highlighted by some participants as a solution to some difficulties to quickly access their doctor, and they could also be used for follow-up consultations, reducing time on the healthcare centres. One participant said,

To have through technology a consultation with the doctor for any symptom I have in order to be more accessible than going to the healthcare centre. I would like to have the possibility of a Videoconsultation (woman, Venezuela, 2 years in Spain).

Additionally, the participants indicated that they would appreciate that the healthcare professionals recommended sites where to find trustworthy information, saying,

We would need a health professional to always recommend reliable websites (woman, Dominican Republic, 20 years in Spain).

#### Educational Needs

Educational provisions in terms of Spanish language, digital skills and how to appropriately search for health information, were expressed by the participants among their needs to increase DHL. They said,

I would like to have more Spanish classes, because if we don't have a problem with the language, it makes everything easier (woman, Dominican Republic, 20 years in Spain).

I signed up for a computer group in “Asturias Acoge” because they see it necessary and I have decided to improve myself (woman, Venezuela, 3–7 months).

We need to know where to look for information, because not everywhere we find the right information (woman, Dominican Republic, 20 years in Spain).

## Discussion

### Main Findings and How They Relate to Previous Findings

Our study highlights the diverse dHL needs and challenges faced by immigrants. Participants view Digital technologies as a useful complement to information from healthcare professionals, mainly for consulting health issues, self-care, verifying information, and improving lifestyles. However, several barriers hinder its effectiveness, including language limitations, low health knowledge, and a lack of confidence in online information.

Participants stressed the importance of accessible, accurate, and practical health information that is free of charge and tailored to specific population groups. Health professionals play a key role in creating culturally sensitive, reliable online health content and recommending trustworthy sources. Strategic community stakeholders also need to be involved in developing health policies and interventions that improve DHL levels.

### Advancing dHL to Address Health Inequalities

Advancing DHL is crucial to prevent inequalities from widening [5]. DHL is a powerful tool to improve health outcomes but much research is still needed on new policies and interventions to improve DHL [4]. This study is intended to provide insights from a qualitative perspective that can inform the development of health policies aimed at increasing DHL in Europe and contribute to the reduction of inequalities affecting immigrant populations.

### Self-Management and Patient Empowerment

Among the results of this study, it has been found that the participants perceive the positive value of DHL in taking a more active role in their own healthcare not only as a preventive measure, gaining knowledge on how to lead healthier lifestyles, but also to improve self-management of the disease and patient empowerment. These results align with previous research which shows that DHL reinforces selfcare and lead to better health outcomes [21, 22].

### Communication Barriers

Language limitations and low levels of education and HL have been pointed out as building blocks for immigrants to better understand medical language and communicate with healthcare professionals, resulting in less patient involvement in healthcare decisions. This is further supported by existing literature which shows the importance of a good communication with clinicians and the positive effect on patients health [23]. Participants pointed to the need for easily understandable information, complemented by resources that enhance content and assist in overcoming linguistic constraints. Failure to use health services effectively or misunderstanding health information can hinder access to vital health information.

### Access to Digital Health Resources

Participants called for easier access to and greater involvement of health professionals in the definition of health information available on the internet. Increased engagement of healthcare providers may have a significant impact on the establishment of medical relationships with cultural sensitivity and the effective management of health, aligning with research that also reflect the need for migrants to feel understood and supported by health professionals [24]. In addition, greater involvement of healthcare professionals would reduce mistrust and reluctance to use digital technologies to obtain health information.

A barrier generally identified in this study was the issue of cost when using certain apps. Even though some studies revealed that the use of digital interventions in healthcare was cost-effective in terms of costs and health outcomes [25], and the fact that all participants use basic digital resources (internet, mobile phones), the cost of healthcare resources still hinders access to health information among immigrants.

### Adaptation of Health Information for Immigrant Populations

Additionally, the adaptation of health-related information to different types of population was required by participants in this study. Adapting health policies to meet the needs of immigrants is crucial for ensuring equitable healthcare access and improving overall public health. Immigrants often face unique challenges, such as language barriers, cultural differences, and limited access to resources, which can hinder their ability to navigate healthcare systems effectively. Policies and interventions should reflect cultural sensitivity and adapt to immigrants’ diversity [26]. It is recommended that strategic stakeholders in the community, providing assistance to immigrants in host countries, be involved in adapting health policies and interventions to help provide targeted-specific sensitivity and improve programme effectiveness. This statement is supported by existing literature that identifies the community stakeholders as strategic for the interventions with vulnerable populations [27–29].

There is an urgent need to strengthen country and region capacities to govern digital transformation in the healthcare sector and advance DHL (2). New policies should pave the way for enabling e-services and developing effective strategies to increase DHL among immigrants and contribute to reducing the digital health divide [5, 30]. Advocacy for person-centred, digitallyenabled health systems will drive DHL and inclusive approaches to digital transformation [31]. Immigrants should be engaged in participatory initiatives addressed to the definition of health policies and the design of health interventions to provide nuances on their specific needs which otherwise may be not perceived by researchers and policymakers, leading to more effective health policies [32].

### Limitations

Although the present study has recruited participants from different countries and periods of time living in Spain, this study could be extended with more participants from other regions of Spain. The limited sample size of 24 individuals and the focus on a single geographic area may constrain the broader applicability of the results. Including data on participants’ age, education and socioeconomic backgrounds and age could have provided additional context for interpretation. Furthermore, recruiting participants primarily through NGOs could have introduced selection bias, potentially influencing the diversity of perspectives captured. The use of field notes in place of full interview transcripts may have affected the depth and nuance of the qualitative data. Moreover, the lack of methodological triangulation—either through complementary qualitative or quantitative approaches—limits the robustness and validation of the findings.

### Conclusions

This study sheds light on the real needs of immigrants to improve their dHL, emphasizing the importance of involving the target populations in the design of technologies, dHL policies and interventions. While barriers related to the inherent characteristics of the immigrant population exist, additional challenges stem from the lack of access to accurate, practical, and easily accessible health information, particularly information that is free of charge. Public authorities, health professionals, and strategic community stakeholders play a crucial role in promoting DHL and improving the health outcomes of immigrant populations. Primary healthcare professionals could play a central role in enhancing DHL among immigrants, as they are consulted more frequently than specialists. This is particularly relevant given the high representation of immigrants in elderly care—where primary care is often the first point of contact—and the reliance of immigrant families with children on these services. Cultural-competency training should be provided for healthcare professionals and digital health providers. Participants view digital health technologies as a powerful tool that empowers them to take a more proactive role in their healthcare, enhancing their ability to manage their health conditions and adopt healthier lifestyles. To enhance DHL among immigrant communities, developing tools with multilingual and interactive interfaces for widely used platforms like Google and YouTube could be highly beneficial. Ensuring these tools meet real needs requires co-design and collaborative strategies that actively involve immigrants in their development.

The findings of this study offer valuable insights for policy and practice, providing policymakers with evidence to develop inclusive strategies that promote dHL and refine health policies tailored to the specific needs of immigrants. However, ongoing monitoring and evaluation of digital health initiatives are essential to ensure their effectiveness, especially for vulnerable populations.

## References

[1] World Health Organization. Global Strategy on Digital Health 2020–2025 (2021). Available online at: https://www.who.int/docs/defaultsource/documents/gs4dhdaa2a9f352b0445bafbc79ca799dce4d.pdf (Accessed January 20, 2025).

[2] Regional Digital Health Action Plan for the WHO European Region 2023–2030 (2022). Available online at: https://iris.who.int/bitstream/handle/10665/360950/72wd05e-DigitalHealth220529.pdf?sequence=2 (Accessed January 20, 2025).

[3] HLS19 Consortium of the WHO Action Network M-POHL. The HLS19-DIGI Instrument to Measure Digital Health Literacy (2022). Available online at: https://m-pohl.net/sites/mpohl.net/files/inline-files/Factsheet%20HLS19-DIGI.pdf (Accessed January 20, 2025).

[4] Arias LMDP Ong BA Borrat Frigola X Fernández AL Hicklent RS Obeles AJT Digital Literacy as a New Determinant of Health: A Scoping Review. PLOS Digit Health (2023) 2:e0000279. 10.1371/journal.pdig.0000279 37824584 PMC10569540

[5] Van Kessel R Wong BLH Clemens T Brand H. Digital Health Literacy as a Super Determinant of Health: More than Simply the Sum of Its Parts. Internet Interv (2022) 27:100500. 10.1016/j.invent.2022.100500 35242586 PMC8861384

[6] Sundell E Wångdahl J Grauman Å. Health Literacy and Digital Health Information-Seeking Behavior – A Cross-Sectional Study Among Highly Educated Swedes. BMC Public Health (2022) 22(1):2278. 10.1186/s12889-022-14751-z 36471284 PMC9724302

[7] Heponiemi T Kaihlanen AM Virtanen L Kainiemi E Saukkonen P Koponen P The Mediating Role of Digital Competence in the Associations Between the Factors Affecting Healthcare Utilization and Access to Care. Int J Public Health (2024) 68:1606184. 10.3389/ijph.2023.1606184 38250321 PMC10796446

[8] World Health Organisation. Digital Health in the European Region: The Ongoing Journey to Commitment and Transformation (2023). Available online at: https://iris.who.int/bitstream/handle/10665/372051/9789289060226eng.pdf?sequence=2&isAllowed=y (Accessed January 20, 2025).

[9] Park S Lee H Kang M. Factors Affecting Health Literacy Among Immigrants - Systematic Review. Eur J Public Health (2018) 28(Suppl. l_4). 10.1093/eurpub/cky214.283

[10] Radu I Scheermesser M Spiess MR Schulze C Händler-Schuster D Pehlke-Milde J. Digital Health for Migrants, Ethnic and Cultural Minorities and the Role of Participatory Development: A Scoping Review. Int J Environ Res Public Health (2023) 20(20):6962. 10.3390/ijerph20206962 37887700 PMC10606156

[11] López-Ventoso M Pisano González M Fernández García C Diez Valcarce I Rey Hidalgo I Rodríguez Nachón MJ Understanding COVID: Collaborative Government Campaign for Citizen Digital Health Literacy in the COVID-19 Pandemic. Life (2023) 13(2):589. 10.3390/life13020589 36836945 PMC9959963

[12] Hernández EE Robles N Angulo-Brunet A Cullen D Del Arco I. Spanish and Catalan Versions of the Ehealth Literacy Questionnaire: Translation, Cross-Cultural Adaptation, and Validation Study. J Med Internet Res (2024) 26:e49227. 10.2196/49227 38728072 PMC11127138

[13] García-García D Ajejas Bazán MJ Pérez-Rivas FJ. Factors Influencing Ehealth Literacy Among Spanish Primary Healthcare Users: Cross-Sectional Study. Int J Environ Res Public Health (2022) 19(23):15497. 10.3390/ijerph192315497 36497572 PMC9738798

[14] IDEAHL Consortium. IDEAHL-improving Digital Empowerment for Active Healthy Living (2022). Available online at: https://cordis.europa.eu/project/id/101057477 (Accessed January 16, 2025).

[15] Pisano González M López-Ventoso M Fernández García C Pruneda González L Rey Hidalgo I IDEAHL Consortium. IDEAHL European Digital Health Literacy Strategy. In: IDEAHL European Digital Health Literacy Strategy. Principado de Asturias. Spain: Zenodo (2024). 10.5281/zenodo.11395540

[16] Bate P Robert G. Experience-Based Design: From Redesigning the System Around the Patient to Co-Designing Services With the Patient. Qual Saf Health Care (2006) 15(5):307–10. 10.1136/qshc.2005.016527 17074863 PMC2565809

[17] Sanders EBN Stappers PJ. Co-Creation and the New Landscapes of Design. CoDesign (2008) 4(1):5–18. 10.1080/15710880701875068

[18] Suprapto N Sunarti T Suliyanah S Wulandari D Hidayaatullaah HN Adam AS A Systematic Review of Photovoice as Participatory Action Research Strategies. Int J Eval Res Educ (2020) 9(3):675. 10.11591/ijere.v9i3.20581

[19] Elo S Kyngäs H. The Qualitative Content Analysis Process. J Adv Nurse (2008) 62(1):107–15. 10.1111/j.1365-2648.2007.04569.x 18352969

[20] MAXQDA. (Berlin, Germany. VERBI Software). (2024). Available online at: https://www.maxqda.com/ (Accessed January 16, 2025).

[21] Gayesa RT Ngai FW Xie YJ. The Effects of Mhealth Interventions on Improving Institutional Delivery and Uptake of Postnatal Care Services in Low-and Lower-Middle-Income Countries: A Systematic Review and meta-analysis. BMC Health Serv Res (2023) 23(1):611. 10.1186/s12913-023-09581-7 37296420 PMC10257264

[22] Fitzpatrick PJ. Improving Health Literacy Using the Power of Digital Communications to Achieve Better Health Outcomes for Patients and Practitioners. Front Digit Health (2023) 5:1264780. 10.3389/fdgth.2023.1264780 38046643 PMC10693297

[23] Anderson HL Moore JE Millar BC. Comparison of Innovative Communication Approaches in Nutrition to Promote and Improve Health Literacy. Ulster Med J (2022) 91(2):85–91. 35722219 PMC9200103

[24] Maia AC Marques MJ Goes AR Gama A Osborne R Dias S. Health Literacy Strengths and Needs Among Migrant Communities From Portuguese-Speaking African Countries in Portugal: A Cross-Sectional Study. Front Public Health (2024) 12:1415588. 10.3389/fpubh.2024.1415588 39022410 PMC11253791

[25] Gentili A Failla G Melnyk A Puleo V Tanna GLD Ricciardi W The cost-effectiveness of Digital Health Interventions: A Systematic Review of the Literature. Front Public Health (2022) 10:787135. 10.3389/fpubh.2022.787135 36033812 PMC9403754

[26] Adigun T Opone E Baidoo B Mathenge M Avoka C Awosiku O. Forgotten and Ignored: Making Digital Health Work for Migrant Population in Africa. Oxf Open Digit Health (2024) 2:oqae023. 10.1093/oodh/oqae023 40230968 PMC11932398

[27] Arturo Alvarez R Marta MPG An LB Raquel VA Delia PM Verushka V Crossing Intersectoral Boundaries to Reach out to Vulnerable Populations with Chronic Conditions in Five European Regions. Arch Community Med Public Health (2021) 182–90. 10.17352/2455-5479.000159

[28] Langer SL Castro FG Chen AC Davis KC Joseph RP Kim W Recruitment and Retention of Underrepresented and Vulnerable Populations to Research. Public Health Nurs (2021) 38(6):1102–15. 10.1111/phn.12943 34240459 PMC8697710

[29] Serrano-Gallardo P Cassetti V Boone ALD Pisano-González MM. Recruiting Participants in Vulnerable Situations: A Qualitative Evaluation of the Recruitment Process in the EFFICHRONIC Study. Int J Environ Res Public Health (2022) 19(17):10765. 10.3390/ijerph191710765 36078487 PMC9518307

[30] Choukou MA Sanchez-Ramirez DC Pol M Uddin M Monnin C Syed-Abdul S. COVID-19 Infodemic and Digital Health Literacy in Vulnerable Populations: A Scoping Review. Digit Health (2022) 8:20552076221076927. 10.1177/20552076221076927 35223076 PMC8874333

[31] World Health Organisation. Global Strategy on Digital Health 2020-2025 (2021). Available online at: https://www.who.int/docs/defaultsource/documents/gs4dhdaa2a9f352b0445bafbc79ca799dce4d.pdf (Accessed January 16, 2025).

[32] Roura M Dias S LeMaster JW MacFarlane A. Participatory Health Research with Migrants: Opportunities, Challenges, and Way Forwards. Health Expect (2021) 24(2):188–97. 10.1111/hex.13201 33528082 PMC8077110

